# Electronic cigarettes: a survey of users

**DOI:** 10.1186/1471-2458-10-231

**Published:** 2010-05-04

**Authors:** Jean-François Etter

**Affiliations:** 1 Institute of Social and Preventive Medicine, Faculty of Medicine, University of Geneva, Switzerland

## Abstract

**Background:**

Little is known about users of electronic cigarettes, or their opinions, satisfaction or how and why they use such products.

**Methods:**

An internet survey of 81 ever-users of ecigarettes in 2009. Participants answered open-ended questions on use of, and opinions about, ecigarettes.

**Results:**

Respondents (73 current and 8 former users) lived in France, Canada, Belgium or Switzerland. Most respondents (77%) were men; 63% were former smokers and 37% were current smokers. They had used e-cigarettes for 100 days (median) and drew 175 puffs per day (median). Participants used the ecigarette either to quit smoking (53 comments), to reduce their cigarette consumption (14 comments), in order not to disturb other people with smoke (20 comments), or in smoke-free places (21 comments). Positive effects reported with ecigarettes included their usefulness to quit smoking, and the benefits of abstinence from smoking (less coughing, improved breathing, better physical fitness). Respondents also enjoyed the flavour of ecigarettes and the sensation of inhalation. Side effects included dryness of the mouth and throat. Respondents complained about the frequent technical failures of ecigarettes and had some concerns about the possible toxicity of the devices and about their future legal status.

**Conclusions:**

Ecigarettes were used mainly to quit smoking, and may be helpful for this purpose, but several respondents were concerned about potential toxicity. There are very few published studies on ecigarettes and research is urgently required, particularly on the efficacy and toxicity of these devices.

## Background

In recent years several manufacturers, mainly in China, have produced electronic cigarettes (ecigarettes) that are distributed in western countries, often by small, newly established companies [1-4]. Electronic cigarettes look and feel like cigarettes, but do not burn tobacco. The several existing brands vary but, in general, ecigarettes contain a battery and an electronic device that produces a warm vapour or 'mist'. The vapour usually contains nicotine and often - but not always - contains propylene glycol [5]. The vapour is inhaled and, as the user exhales, some visible vapour is released, but no tobacco smoke. Some ecigarettes also contain a light-emitting diode in the tip that glows when the user puffs, to resemble the burning end of a cigarette. The nicotine content of the cartridge varies, and the cartridges usually contain chemical additives and flavours (such as various brands of tobacco, chocolate, coffee, mint or fruit). The cartridges can usually be refilled, and refill bottles are provided with the device.

Electronic cigarettes are probably less harmful than tobacco smoking, but they are almost certainly more dangerous than medicinal nicotine inhalers [6,7]. However, to our knowledge, there is no published data on the safety of ecigarettes. Internationally, the legality of ecigarettes varies; they cannot be sold in Australia, Brazil, Canada, Denmark or Switzerland, but their sale is authorized in other countries (e.g. China, New Zealand) [5,8,9]. Analyses conducted by the United States Food and Drug Administration (FDA) showed that ecigarettes contain carcinogens, including nitrosamines, toxic chemicals such as diethylene glycol, and tobacco-specific components suspected of being harmful to humans (anabasine, myosmine, and beta-nicotyrine) [6]. The FDA also found that ecigarette cartridges labelled as containing no nicotine did in fact contain low levels of nicotine. Some manufacturers do not disclose the ingredients in their products. Furthermore, ecigarettes are not manufactured according to the high standards imposed on pharmaceutical companies. Consequently, the inhaled vapour may contain impurities that may be dangerous to consumers [6]. In particular, the origin of the nicotine itself is uncertain, as pesticide-grade nicotine rather than pharmacological-grade nicotine may be used in ecigarettes.

Little is known about ecigarettes, as few research reports have been published [10,11]. In addition to the FDA report mentioned above, reports from New Zealand, funded by Ruyan (a Chinese manufacturer of ecigarettes) concluded that the mist from the Ruyan ecigarette contains acetaldehyde and mercury [12,13]. A randomised trial in 40 smokers found that the Ruyan ecigarette delivered nicotine to the blood more rapidly than the nicotine inhaler, but less rapidly than cigarettes, and that the effect of the ecigarette on craving was similar to that of the nicotine inhaler, but less than that of cigarettes [14]. A recent U.S. study found that 10 puffs of an ecigarette delivered little or no nicotine [15].

The mist from ecigarettes is inhaled into the lung [13]. Although the particle size is apparently too small to ensure deposition in the lung alveoli [12], we are not aware of any study of lung absorption of ecigarette mist. Because lung inhalation may enable nicotine to pass rapidly into the blood, and thus rapidly relieve craving and tobacco withdrawal symptoms [14], ecigarettes have the potential to be at least as effective as currently approved nicotine replacement therapy (NRT) products, none of which deliver nicotine to the lung. In addition, the similarities in shape, actions and inhalation between ecigarettes and tobacco cigarettes could also help smokers quit. However, as there are no data to support the manufacturers' claims that ecigarettes help smokers quit, the World Health Organization asked the companies not to make any therapeutic claims [7,16]. If they claimed that ecigarettes help smokers quit, manufacturers would be subject to the legislation and regulation that applies to NRT products. In order to avoid this, some ecigarettes are now marketed for enjoyment, or as devices that enable smokers to "smoke" everywhere, including smoke-free places [3,4]. Nonetheless, some distributors present their products as an alternative to tobacco smoking, more or less implicitly suggesting that ecigarettes can be used to aid smoking cessation [1,2].

One may hypothesize that the positive effects of ecigarettes may include smoking cessation, smoking reduction or relapse prevention. The ecigarette could also be used as an aid during a preparation period before cessation, similar to the pre-cessation treatment or "cut down to quit" approach that is an approved indication for NRT [17]. On the other hand, ecigarettes may be dangerous because of the frequent and longterm lung inhalation of diethylene glycol, nicotine and other toxic components, and because of the sub-standard manufacturing process, relative to pharmaceutical products [7]. Because of its rapid nicotine delivery [14], the ecigarette also has the potential to be addictive. In addition, the refill bottles may be dangerous as they contain up to one gram of nicotine, whereas the fatal dose of nicotine is estimated to be 30 to 60 mg for adults and 10 mg for children [5]. The ecigarette may also enable smokers to continue to 'smoke' in smoke-free environments, thus delaying or preventing cessation in people who might otherwise quit. Finally, the fruit and chocolate flavours may appeal to young people, and this raises the concern that ecigarettes may facilitate initiation of nicotine dependence in young never-smokers [5]. However, none of these hypotheses has yet been tested.

Because of the huge burden of tobacco-related death and disease, and because ecigarettes have potential to help smokers quit, there is an urgent need for research into these products. First, there is a need to know why and how these products are used, and whether users are satisfied with them. The aim of this study was to assess usage patterns of ecigarettes, reasons for use, and users' opinions of these products.

## Methods

As ecigarettes are mainly sold online, the internet is a logical way to reach users. We therefore posted a survey form, in French, on the smoking cessation website StopTabac.ch over a 34 day period between September and October 2009. This website receives approximately 120,000 visitors per month and is principally visited either by smokers who intend to quit or by recent quitters [18,19]. Links to the survey were posted on websites that either provide information about ecigarettes (ecigmag.com, forumecigarette.com) or sell them (econoclope.com, sedansa.be). After discussion with the head of the ethics committee of the Geneva University Hospitals (community medicine section, the committee to which our Institute is submitted), the study was exempted from approval.

Eligible participants were people who declared that they had ever used an ecigarette and who provided the brand name of the ecigarette that they had used most often. Subjects who did not name a brand were excluded, because this raised doubts about whether they had actually used an ecigarette. On the survey form, participants indicated whether they had ever used ecigarettes or were currently using them (subdivided into daily user, non-daily user, former user, never used). They also provided the total number of days that they had been using ecigarettes, the brand they used most often, the nicotine dose per unit, the flavour and the cost per package (using open-ended questions). In addition, subjects indicated whether ecigarettes had helped them to quit smoking, and current users indicated the number of puffs per day on ecigarettes.

In response to open-ended questions, participants wrote where they bought their ecigarettes, the reasons why they used them, what they considered to be the beneficial and undesirable effects of ecigarettes, and the most positive and negative points about the product. If they had stopped using ecigarettes, they explained why. Participants also listed which questions they had asked themselves about ecigarettes, and gave their opinion on the information leaflets or documents inserted in the ecigarette packages. Finally, they wrote general comments on the ecigarette.

Other questions also covered smoking status (daily, non-daily, former smoker, never smoker). Smokers stated the number of cigarettes they smoked per day, and former smokers stated when they had quit smoking. Participants were asked to supply their age, sex and country of residence.

Medians rather than means were used for continuous variables because medians are less sensitive to outliers, which can excessively influence means in small sample sizes.

## Results

Answers were obtained from 214 people, but 123 of these had never used ecigarettes and ten did not name the brand of their ecigarette. These 133 subjects were excluded. All subsequent analyses included only the 81 respondents who declared that they had ever used ecigarettes and who indicated the brand that they had used most often. These 81 respondents included 72 daily users of ecigarettes, one non-daily user and eight former users (Table 1). They were relatively young (median age 37 years), and most (77%) were men. Respondents lived in France (81%), Belgium (8%), Canada (6%) and Switzerland (5%). Most (63%) were former smokers who had quit smoking relatively recently (median duration of abstinence: 100 days) (Table 1).

**Table 1:** Characteristics of ecigarette users, and usage patterns

*Characteristic*	
Number of respondents	81
Age, median (range), years	37 (19-65)
Men (%)	77
Smoking status (%)	
Former smokers	63
Daily smokers	23
Occasional (non-daily) smokers	13
Cigarettes per day, in smokers (median)	12
Days of abstinence, in former smokers, median (25^th ^and 75^th ^percentiles)	100 (30, 210)
	
*Use of electronic cigarettes*	
Days of use of the e-cigarette, median (25^th ^and 75^th ^percentiles)	100 (30, 210)
Number of puffs per day, median (25^th ^and 75^th ^percentiles)	175 (90, 275)
Number of puffs per day, range	10 to 600
Price per package, median, Euros (U.S. dollars)	40 (60)
Median dose of nicotine per unit, mg (25^th ^and 75^th ^percentiles)	14 (10, 16)
Does (did) the e-cigarette help you quit smoking? (%)	
Yes, a lot	79
Yes, somewhat	16
No, not at all	5

### Use of the electronic cigarette

Most respondents had been using the ecigarette for slightly longer than three months, and current users took 175 puffs per day (median) from their device (Table 1). Sixteen different brands of ecigarettes were named, the most frequent being Janty (n = 17), Joye (n = 17), Sedansa (n = 14), Econoclope (n = 9), Liberty-cig (n = 8), Smoke51 and Edsylver (n = 2 each). All these brands of ecigarette deliver nicotine, and the median dose of nicotine per unit was 14 mg. The preferred flavour (open-ended field, 78 answers) was tobacco (n = 46, various flavours, e.g. "Turkish blend", "K-mel"), followed by mint (n = 6), fruit (n = 5, e.g. "apple"), vanilla (n = 4), coffee (n = 3) and tea (n = 2). Twelve respondents used several of these flavours.

Most respondents (n = 74; 94% of 79 answers) had bought their ecigarette on the internet, two had bought their device in China, two at a tobacco retail shop and one had bought it second hand. When asked whether the ecigarette helped them quit smoking, most respondents (79%) answered "a lot" (Table 1).

When asked why they chose to use ecigarettes (three open-ended fields, 225 comments), the most frequent answers were: that they used it to quit smoking; for their health (as ecigarettes were perceived to be less toxic than tobacco, e.g.: "it is better for health than tobacco"); because ecigarettes are less expensive than regular cigarettes; because ecigarettes can be smoked everywhere, including smoke-free places (e.g.: "I don't need to go outside to smoke anymore"); to avoid disturbing other people with second-hand smoke; for the pleasure of smoking it (e.g.: "to continue to inhale, which is something I like"), and to reduce their cigarette consumption (Table 2).

**Table 2:** Reasons for using e-cigarettes: open-ended comments from e-cigarette users

	Number of comments
To quit smoking	53
For health, as e-cigarettes were perceived to be less toxic than tobacco	49
Less expensive than regular cigarettes	26
Can be smoked everywhere, including smoke-free places	21
To avoid disturbing other people, or producing environmental tobacco smoke or the smell of stale smoke	20
For the pleasure of smoking, including the pleasure of inhaling and smoking-related actions	19
To reduce cigarette consumption	14
Curious to test a new product	10
Ecigarettes taste and smell good	8
Previously failed to quit with either nicotine patch or bupropion	3
To get nicotine	2
*Total (from three open-ended fields)*	*225*

The most frequently cited beneficial effects of ecigarettes (two open-ended fields, 134 comments) were: that it improved breathing and respiration (e.g.: "I have less breathlessness on exertion"); that it helps to quit smoking (e.g.: "I have quit smoking without problems"); that respondents coughed less, expectorated less and had fewer sore throats; that it improved their health and physical fitness; and that it did not cause unpleasant odours or bad breath (Table 3). Interestingly, one respondent suggested that the ecigarette device might be useful to administer other medications to the bronchia or lung. The two open-ended fields on the undesirable effects of ecigarettes elicited 61 comments (only half the number of comments received on the beneficial effects). The most frequent responses were that ecigarettes caused dry mouth and throat, vertigo, headache or nausea (Table 3).

**Table 3:** Beneficial and undesirable effects of e-cigarettes: open-ended comments from ecigarette users

	Number of comments
*Beneficial effects (total from two open-ended fields)*	*134*
Improves breathing and respiration	31
Less cough, less expectoration, fewer sore throats	23
Helps to quit smoking	20
Improves health and physical fitness	17
Improves sense of taste and smell	11
Does not cause unpleasant odours or bad breath	10
Helps to reduce cigarette consumption	7
Sleeps better	4
Less craving for cigarettes	4
Cost	4
Pleasure of smoking the e-cigarette	2
Useful device to administer other medications to the bronchia or lung	1
	
*Undesirable effects (total from two open-ended fields)*	*61*
Dry mouth and throat	16
Vertigo, headache or nausea	7
Bad taste	4
Weight gain	3
Technical problems (batteries)	3
Difficult to accurately control dose of nicotine	3
Cost	3
No undesirable effects	13
Miscellaneous comments	9

The most frequently cited positive features of ecigarettes (three open-ended fields, 208 comments) were: that respondents liked the taste and variety of flavours; they appreciated the beneficial effects of the ecigarette on their health, breathing and cough; the absence of unpleasant odours or bad breath; they appreciated the pleasure of inhalation, and harsh sensation in the throat; they liked the act of using the ecigarette, which is similar to smoking; the ecigarette is less toxic than tobacco smoke; it facilitates smoking cessation; and that it can be used everywhere (Table 4).

**Table 4:** The most positive and negative aspects of ecigarettes: open-ended comments from e-cigarette users

	Number of comments
*Positive points (total from three open-ended fields)*	*208*
Taste and variety of flavours	38
Beneficial effects on health, breathing and cough	26
No unpleasant odours or bad breath	23
Inhalation, including harsh sensation in the throat and pleasure of inhaling	16
Less toxic than tobacco smoke	15
Facilitates smoking cessation	15
Can be used everywhere (the freedom)	15
The gestures or actions (similar to smoking)	13
Ease of use, design	10
Less expensive than cigarettes	9
No environmental tobacco smoke	8
Facilitates smoking reduction	5
No ash, dirt, or burned clothes	5
Can choose the dose of nicotine and number of puffs	5
Relieves craving for tobacco	3
Improves sense of smell and taste	2
	
*Negative points (total from three open-ended fields)*	*154*
Poor quality, lack of reliability and frequent failures	40
Batteries discharge too rapidly	27
Too expensive	14
Bad taste	14
Difficult or impractical to use; dosage is difficult to adjust	10
The liquid may leak during usage	10
Only sold on the internet	9
No studies or information on the composition of the vapour and the health risks of the e-cigarette	8
Cartridges do not last long enough	6
Difficult to stop using the ecigarette without relapsing to smoking	4
Too big or too heavy	3
Too often asked by friends or colleagues to explain the device	2
Miscellaneous	7

When asked about the three most negative aspects of ecigarettes (three fields, 154 comments), respondents complained in particular about the poor quality of the devices. They also reported that that ecigarettes were difficult or impractical to use (e.g. "it is difficult to refill the liquid"), that the dosage was difficult to adjust (either too high or too low), that the liquid can leak out during use, and complained about the lack of information on the composition of the vapour and any health risks associated with ecigarettes (Table 4).

Respondents also stated which questions they had asked themselves about ecigarettes (three fields, 112 comments). This section showed that users wondered whether ecigarettes were safe, what the effects on health were, and whether ecigarettes are toxic (59 comments, including five that specifically mentioned propylene glycol). Respondents were also concerned that the e-cigarette might be banned, and about its future legal status (19 comments, e.g.: "let's hope it will not be prohibited"). They wanted to know about the composition of the liquid in the cartridge (10 comments, e.g.: "What exactly is the content of this liquid?", including four comments on the quality of the liquids), why no serious studies on ecigarettes have been published (5 comments), why ecigarettes are not sold in pharmacies (4 comments) and why the devices are not produced in western countries (3 comments).

When asked to comment on the documentation that accompanied their ecigarette (one field, 70 comments), most respondents answered that the inserts were good or satisfactory (31 comments), seven responded that they were only adequate, 15 responded that they contained too little information, four reported that there was no explanatory leaflet with their ecigarette, and two complained that there was no explanation of the health effects of ecigarettes. Three people responded that they used the internet and online discussion forums to obtain more information on ecigarettes (e.g.: "the insert was very brief, but fortunately, there are specialized internet discussion forums").

The section that asked participants to write general comments on the ecigarette (one field) elicited 64 comments. Twenty-one comments were very positive or enthusiastic (e.g. "brilliant" (6 times), "miracle product", "unbelievable", "very satisfied"), and 11 were positive but more neutral (e.g.: "good", "I recommend it"). Respondents also considered that the ecigarette helped them quit smoking (14 comments), that it was more effective than either nicotine patch or bupropion (5 comments), and that it enabled them to reduce their cigarette consumption (3 comments). Three people feared that the ecigarette would soon be banned. Four commented that ecigarettes need technical improvement, and six wrote negative comments (e.g.: "not helpful to quit", "avoid it").

Eight respondents had stopped using ecigarettes, and were asked to indicate why (two fields, 15 comments). Reasons included: it did not help me quit smoking (6 comments); it did not taste like cigarettes (3 comments); poor quality or not reliable (3 comments); because of concerns about risks and side-effects of ecigarettes (3 comments).

Interestingly, several respondents used a neologism (*vapoter*, in French) to describe the action of smoking an ecigarette; this term probably originated from "vapour" and spread in online discussion forums. The corresponding terms used on English-language forums (e.g. ecigarette-forum.com) are "vaping" and "vaper".

## Discussion

Although, for legal reasons, ecigarettes are mainly marketed to current smokers either for enjoyment or for use in smoke-free places, our results suggest that most people who buy these products are current and former smokers who use ecigarettes to help quit smoking, just as they would use NRT. Our survey also showed that ecigarettes were liked by users, and were used quite intensively by this sample; almost all respondents were daily ecigarette users, and the number puffs per day (175) was substantial. However, as ecigarettes deliver about one-tenth of the nicotine per puff compared to cigarettes [12], this intensive puffing pattern may result in less exposure to nicotine than smoking. Interestingly, the median duration of ecigarette use corresponded to the median duration of abstinence in former smokers (100 days in both cases).

Respondents reported more positive than negative effects with ecigarettes: many reported positive effects on the respiratory system (breathing better, coughing less), which were probably associated with stopping smoking [20]. The fact that ecigarettes do not produce any unpleasant odours or environmental tobacco smoke was also appreciated. Most importantly, many respondents reported that the ecigarette helped them quit smoking, and several compared it favourably with either nicotine patch or bupropion. These preliminary findings, together with data showing that ecigarettes relieve craving and withdrawal [14], suggest that the ecigarette may be an effective aid to smoking cessation, and therefore merits serious investigation for this purpose. Ideally, future trials should compare the efficacy of ecigarettes versus NRT (particularly the nicotine inhaler), bupropion or varenicline. However, as ecigarettes are probably more toxic than NRT products [6], the former should probably only be recommended to smokers if they are substantially more effective than current NRTs, and if the toxic constituents of ecigarettes can be eliminated.

Interestingly, dry mouth and throat was a frequent adverse effect of the ecigarette. It may be useful to investigate why this occurs and how it might be minimised. It would also be interesting to investigate why ecigarettes appeal more to men than to women. Many respondents complained of the poor quality of ecigarettes, their frequent failures, the lack of durability of cartridges and batteries, and that the liquid sometimes leaks from the device during usage. Apparently competition between manufacturers has not yet resulted in products of sufficient technical quality.

Although users' comments were generally positive, many were concerned about the safety and toxicity of ecigarettes, and questioned why no study has yet investigated these aspects. Several respondents were also concerned about the future legal status of ecigarettes, and that they may possibly be banned. Indeed, health authorities in several countries have published warnings about, or have prohibited the sale of, ecigarettes [5-8]. From a public health perspective, however, the question is whether - at a population level - the potential benefits of the ecigarette outweigh its drawbacks. If ecigarettes are more effective than current NRTs, but are withdrawn from the market until approved as smoking cessation aids, ecigarette users might revert to smoking tobacco, which is more hazardous than ecigarettes. This could have a significant, negative impact on public health, because it can take several years to obtain legal approval for a new drug delivery system.

On the other hand, ecigarettes are not currently manufactured to the same rigorous standards as pharmaceutical products; they currently contain toxic components and are therefore almost certainly less safe than NRT products [6]. The legal status of the e-cigarette is unclear in many countries, and its regulation is complex; it is neither classed as a tobacco product, nor food, nor is it registered as a medicine. From the legal perspective, there is a difficult balance between the need to protect consumers and the possibility now being offered to smokers to use a new, acceptable and potentially effective device to stop smoking. Given the enormous burden of disease and death caused by tobacco smoking, there is an urgent need for research into the toxicity, efficacy and public health impact of ecigarettes [10]. In addition, whether devices that resemble ecigarettes could be used to deliver medications other than nicotine to the lung and bronchia also warrants investigation. As the manufacturers and distributors of ecigarettes are relatively small companies that may be unable to afford the research costs, or possess the expertise or manpower to go through the regulatory approval process, support from governments, public health organizations or foundations may be needed to produce evidence on these novel devices.

One limitation of our study is that it was conducted in a self-selected sample of internet users. Whether this method over-sampled satisfied users, long-term users or heavy users of ecigarettes is unknown. Compared to population-based samples of smokers in Europe or the United States, visitors to the Stop-Tabac.ch website are more likely to have made a quit attempt in the previous year, are more motivated to quit smoking, are slightly less dependent on tobacco, and are more highly educated [18,19]. Thus, although our results provide useful and interesting preliminary information on ecigarette users, our findings may not be generalizable and should be interpreted with caution.

## Conclusions

Our results suggest that ecigarettes are used mainly to quit smoking, and may be useful for this purpose. However, users were concerned about the potential toxicity of these devices. Very few studies have investigated ecigarettes and research is now urgently required, particularly to establish the efficacy and toxicity of these devices.

## Competing interests

The Institute of Social and Preventive Medicine of the University of Geneva received trial medications in 2005 from Pfizer, and the author consulted for Pfizer, a manufacturer of smoking cessation medications, in 2006-2007 (on the Swiss varenicline advisory board). No competing interest since then. No link to companies that either produce or distribute ecigarettes.

## Funding

No external funding

## Pre-publication history

The pre-publication history for this paper can be accessed here:

http://www.biomedcentral.com/1471-2458/10/231/prepub
